# Vitamin D Receptor Deficiency and Low Vitamin D Diet Stimulate Aortic Calcification and Osteogenic Key Factor Expression in Mice

**DOI:** 10.1371/journal.pone.0035316

**Published:** 2012-04-20

**Authors:** Nadine Schmidt, Corinna Brandsch, Hagen Kühne, Alexandra Thiele, Frank Hirche, Gabriele I. Stangl

**Affiliations:** Institute of Agricultural and Nutritional Sciences, Martin-Luther-University Halle-Wittenberg, Halle (Saale), Germany; Harvard Medical School, United States of America

## Abstract

Low levels of 25-hydroxy vitamin D (25(OH)D) are associated with cardiovascular diseases. Herein, we tested the hypothesis that vitamin D deficiency could be a causal factor in atherosclerotic vascular changes and vascular calcification. Aortic root sections of vitamin D receptor knockout (VDR^−/−^) mice that were stained for vascular calcification and immunostained for osteoblastic differentiation factors showed more calcified areas and a higher expression of the osteogenic key factors Msx2, Bmp2, and Runx2 than the wild-type mice (P<0.01). Data from LDL receptor knockout (LDLR^−/−^) mice that were fed western diet with either low (50 IU/kg), recommended (1,000 IU/kg), or high (10,000 IU/kg) amounts of vitamin D_3_ over 16 weeks revealed increasing plasma concentrations of 25(OH)D (P<0.001) with increasing intake of vitamin D, whereas levels of calcium and phosphorus in plasma and femur were not influenced by the dietary treatment. Mice treated with the low vitamin D diet had more calcified lesions and a higher expression of Msx2, Bmp2, and Runx2 in aortic roots than mice fed recommended or high amounts of vitamin D (P<0.001). Taken together, these findings indicate vitamin D deficiency as a risk factor for aortic valve and aortic vessel calcification and a stimulator of osteogenic key factor expression in these vascular areas.

## Introduction

Vitamin D deficiency has become a widespread public health problem [1]. Humans normally get vitamin D from sunlight exposure, from their diets or dietary supplements. Seasonal variations of ultraviolet (UV)-B light exposure, increasing indoor activities, and the frequent use of sunscreens are reasons for the low synthesis of cutaneous vitamin D. These people strongly depend on dietary sources of vitamin D. Recent data, however, revealed that the prevalence of inadequate vitamin D intake is very high in large parts of Europe [2].

The classical role of calcitriol (1,25(OH)_2_D_3_), the bioactive form of vitamin D_3_, is to maintain calcium homeostasis and to promote bone mineralisation [3]. However, a growing body of evidence suggests that vitamin D could also modify the risk for cardiovascular diseases. Several longitudinal observational cohort analyses found low plasma concentrations of 25(OH)D associated with an increased risk for incident of cardiovascular diseases [4]–[8] and observational studies found reduced cardiovascular mortality in patients with chronic kidney disease that received calcitriol [9], [10]. Since atherosclerotic degeneration of blood vessels is often the basic underlying pathology of cardiovascular outcomes, the question arises whether vitamin D would have any causal role in pathological vascular changes. Compared to the huge number of association studies, there have been only few studies on the mechanistic effects of vitamin D deficiency on the vascular wall. Recent data from vitamin D receptor knockout (VDR^−/−^) mice, an animal model that emulates vitamin D deficiency [11], revealed a far greater number of fibrotic lesions in the hearts of these mice compared to those of the wild-type mice [12]. However, aortic valves and blood vessels of VDR^−/−^ mice had not been investigated in this study. In a mouse model of chronic kidney disease, activators of vitamin D receptor such as calcitriol and paricalcitrol were effective in protection against aortic calcification in dosages that normalize secondary hyperparathyoidism, whilst higher dosages stimulated aortic calcification [13]. In 2010, Takeda et al. demonstrated that orally administered calcitriol reduced atherosclerotic lesion formation in ApoE null mice [14]. To our knowledge, the physiological impact of a lack of vitamin D, and particularly a lack of its native form, as reflected by the 25-hydroxy-vitamin D (25OHD) on vascular composition and atherosclerotic plaque development remains largely unexplored.

The aim of the present work was to investigate aortic valve and vessel composition in response to VDR deficiency by use of a VDR^−/−^ mouse model. Since, we were able to show, for the first time, that VDR^−/−^ mice compared to wild-type mice showed detectable numbers of calcified spots in their aortic valves, accompanied by an up-regulation of osteogenic key factors, we wanted to examine whether the observable changes in calcification are also seen in animals fed a low vitamin D diet. Therefore a second experiment was conducted with LDL receptor knockout (LDLR^−/−^) mice that were investigated for aortic vessel composition and protein expression in response to a low vitamin D diet and diets that contain recommended or high amounts of vitamin D.

## Results

### Study 1

#### Impact of VDR deficiency on body weight and blood vessel composition

**Figure 1:**
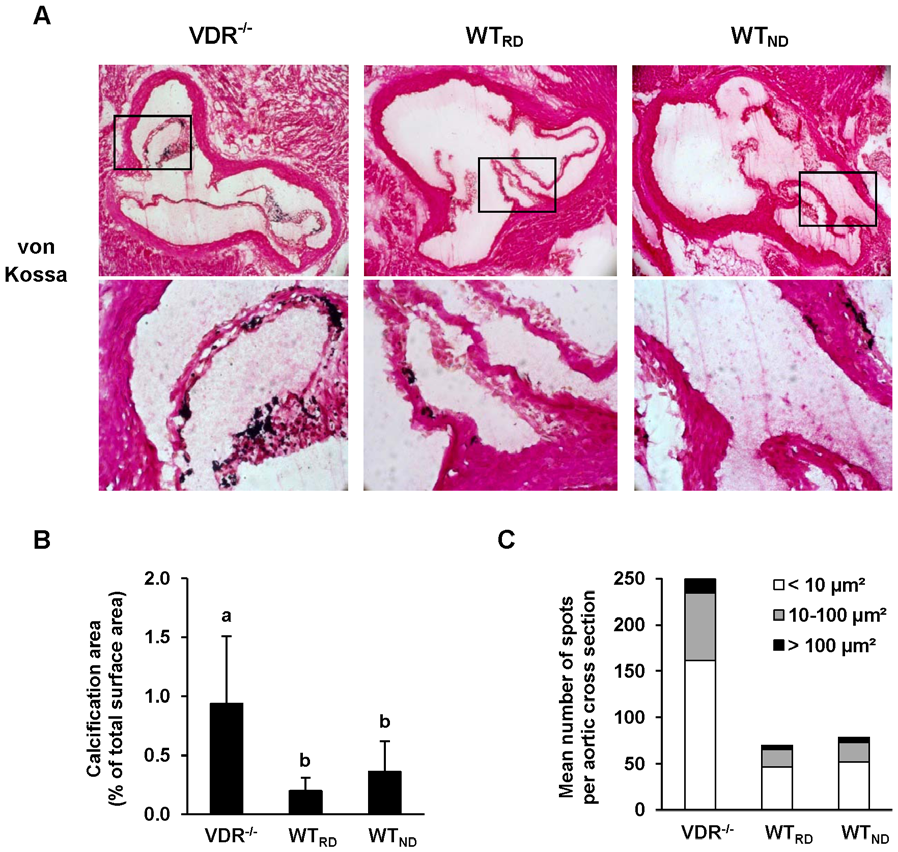
VDR^−/−^ mice have larger calcified lesion areas in their aortic valves than WT mice. Panel A shows representative von Kossa stained aortic root sections of VDR^−/−^ mice fed a rescue diet, WT mice fed a rescue diet (WT_RD_) and WT mice fed a normal calcium/phosphorus diet (WT_ND_) for 8 weeks (×10). The bottom panel shows a magnification of the inserts in the top panel. Panel B shows the relative calcification area of the aortic sections and panel C the number of small-, medium- and large-sized calcified areas within an aortic cross-section. Data are means ± SD of 6–9 mice per group. ^a,b^ Bars with different superscript letters differ, *P*<0.015.

At the end of the feeding period the VDR^−/−^ mice weighed less than the WT mice (VDR^−/−^: 22.9±3.3 g, WT_RD_: 26.1±1.5 g, WT_ND_: 25.2±1.5 g; *P*<0.05). Analysis of aortic root cross-sections revealed no detectable atherosclerotic lesions in any of the mice. During preparation and dissection of the blood vessels we noticed that aortas of VDR^−/−^ mice had been more fragile than those of the WT mice. Von Kossa staining revealed that calcification areas of aortic valves of VDR^−/−^ mice were 4.7 times greater than those of the WT_RD_ mice, and 2.6 times greater than those of the WT_ND_ mice (*P*<0.001; Figure 1). No significant differences in calcification areas were observed between the two groups of WT mice. Figure 1C demonstrate that VDR^−/−^ mice had a markedly higher number of calcified spots within the aortic cross section than WT mice. The increased number of calcified spots was comprised by a higher number of small (<10 µm^2^), medium (10–100 µm^2^) and large (>100 µm^2^) spots, respectively, compared to WT mice. The total number of calcified spots and the number of small, medium and large-sized spots were comparable between the two WT groups.

#### Impact of VDR deficiency on osteoblast differentiation factors in aortic roots

**Figure 2:**
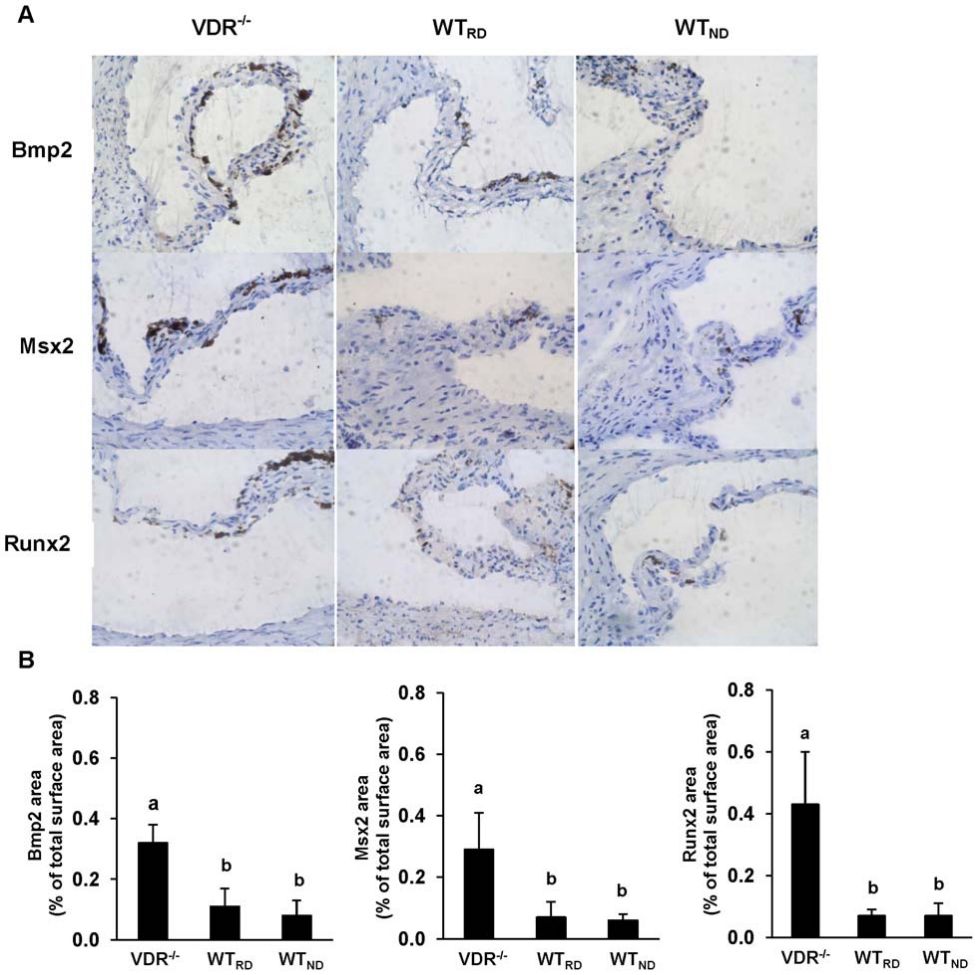
VDR knockout increases the expression of osteoblast differentiation factors in aortic valves. Panel A shows representative aortic root sections immunohistochemically stained for Bmp2, Msx2 and Runx2 from VDR^−/−^ mice fed a rescue diet, WT mice fed a rescue diet (WT_RD_) and WT mice fed a normal calcium/phosphorus diet (WT_ND_) for 8 weeks (×10). Panel B shows the percentage of stained areas relative to the total cross-section. Data are means ± SD of 6–9 mice per group. ^a,b^ Bars with different superscript letters differ, *P*<0.015.

To gain insight into the mechanisms underlying the effect of VDR deficiency on vascular calcification, aortic valve sections were immunohistochemically characterised for osteoblast differentiation factors. As shown in Figure 2, VDR^−/−^ mice had 3 to 6 times higher protein levels of Bmp2 (*P*<0.001), Msx2 (*P*<0.001), and Runx2 (*P*<0.001) than WT mice. No differences in Bmp2, Msx2, and Runx2 were observed between WT_RD_ and WT_ND_ mice, respectively (Figure 2).

Plasma concentration of calcium was lower in the VDR^−/−^ mice than in the WT mice (VDR^−/−^: 1.77±0.24 mmol/L, WT_RD_: 2.28±0.04 mmol/L, WT_ND_: 2.30±0.07 mmol/L; *P*<0.001). Circulating concentration of total phosphorus (organic and inorganic) in the VDR^−/−^ group was higher than in the WT group fed the normal calcium diet (VDR^−/−^: 6.37±0.25 mmol/L, WT_RD_: 6.12±0.44 mmol/L, WT_ND_: 5.62±0.67 mmol/L; *P*<0.05). However, plasma concentrations of inorganic phosphate were not different between the three groups of mice (VDR^−/−^: 1.69±0.26 mmol/L, WT_RD_: 1.63±0.32 mmol/L, WT_ND_: 1.69±0.19 mmol/L).

### Study 2

#### Impact of vitamin D deficiency on body weight, plasma lipids and vitamin D status of the LDLR−/− mice

**Figure 3:**
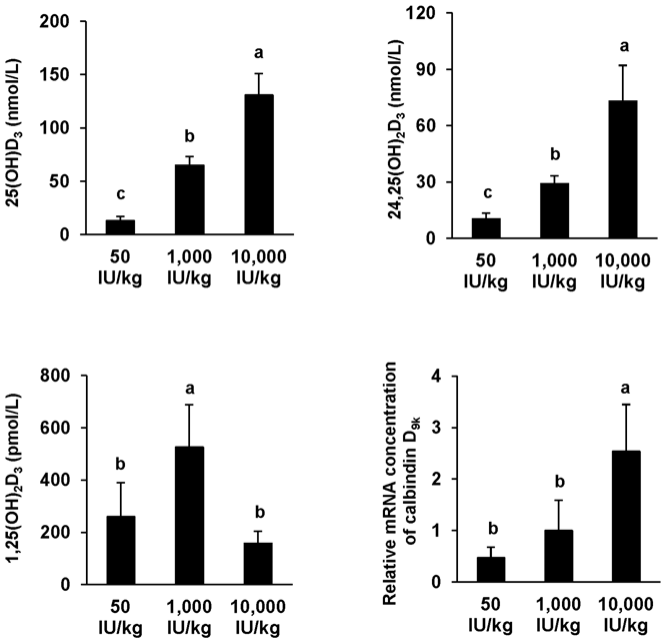
Vitamin D status of LDLR^−/−^ mice in response dietary vitamin D levels. Plasma concentrations of 25(OH)D_3_, 24,25(OH)_2_D_3_, 1,25(OH)_2_D_3_, and relative mRNA abundancy of aortic calbindin D_9 k_ of LDLR^−/−^ mice fed a *Western* diet with low (50 IU/kg), recommended (1,000 IU/kg), or high (10,000 IU/kg) levels of vitamin D_3_ for 16 weeks. Data are means ± SD of 4 probes per group (pools of 3 samples) for analysis of 1,25(OH)_2_D_3_ and 12 mice per group for all other analyses. ^a,b,c^ Bars with different superscript letters differ, *P*<0.05.

The body weights of LDLR^−/−^ mice that were fed diets with low, recommended or high levels of vitamin D_3_ for 16 weeks did not differ (Table 1). The plasma concentrations of triglycerides and cholesterol were also not different between the three groups of mice (Table 1). As expected, the vitamin D status of mice, determined as plasma concentrations of 25(OH)D_3_, 24,25(OH)_2_D_3_, and 1,25(OH)_2_D_3_ was strongly influenced by the vitamin D_3_ concentrations in the diets (Figure 3; *P*<0.001). Mice fed the low vitamin D diet had the lowest plasma concentrations of 25(OH)D_3_ and 24,25(OH)_2_D_3_, followed by mice that received recommended amounts of vitamin D; the highest plasma levels were observed in the high vitamin D group. Mice fed the diets with low and high concentrations of vitamin D had suppressed levels of circulating 1,25(OH)_2_D_3_ compared to mice fed a diet that contained recommended amounts of vitamin D (Figure 3, *P*<0.05). To gain information about the intracellular genomic effects of vitamin D in response to the vitamin D diet, the aortic mRNA abundancy of calbindin D_9 k_ as a classical target gene of the bioactive vitamin D was measured. The relative mRNA concentration of calbindin D_9 k_ in the aorta increased with increasing dietary intake of vitamin D (Figure 3, *P*<0.05). The differences in the vitamin D status, however, were not accompanied by any differences in the concentrations of calcium, total or inorganic phosphate in plasma and femur (Table 2).

**Table 1:** Final body weights and plasma lipid levels of LDLR^−/−^ mice in fed low, recommended or high amounts of vitamin D_3_ with their diets.

	Dietary vitamin D_3_ concentrations		
	50 IU/kg	1,000 IU/kg	10,000 IU/kg
Final body weight (g)	28.5±2.1	27.9±2.9	26.8±2.2
Plasma triglycerides (mmol/L)	1.83±0.68	2.00±0.70	1.85±0.86
Plasma cholesterol (mmol/L)	29.3±4.6	28.3±4.7	28.1±7.0

Values are means ± SD (n = 12 per group).

**Table 2:** Calcium and phosphorus concentrations of plasma and femur of LDLR^−/−^ mice in fed low, recommended or high amounts of vitamin D_3_ with their diets.

	Dietary vitamin D_3_ concentrations		
	50 IU/kg	1,000 IU/kg	10,000 IU/kg
Plasma			
Calcium (mmol/L)	1.93±0.20	1.99±0.21	1.85±0.26
Total phosphorus (mmol/L)	11.5±1.1	11.8±1.4	11.9±2.0
Inorganic phosphate (mmol/L)	4.20±1.00	4.56±1.38	4.38±1.59
Femur			
Calcium (g/kg DM)	235±12	237±5	236±5
Total phosphorus (g/kg DM)	114±6	115±2	114±2

Values are means ± SD (n = 12 per group); DM, dry matter.

#### Impact of a low vitamin D diet on composition of aortic roots of the LDLR−/− mice

**Figure 4:**
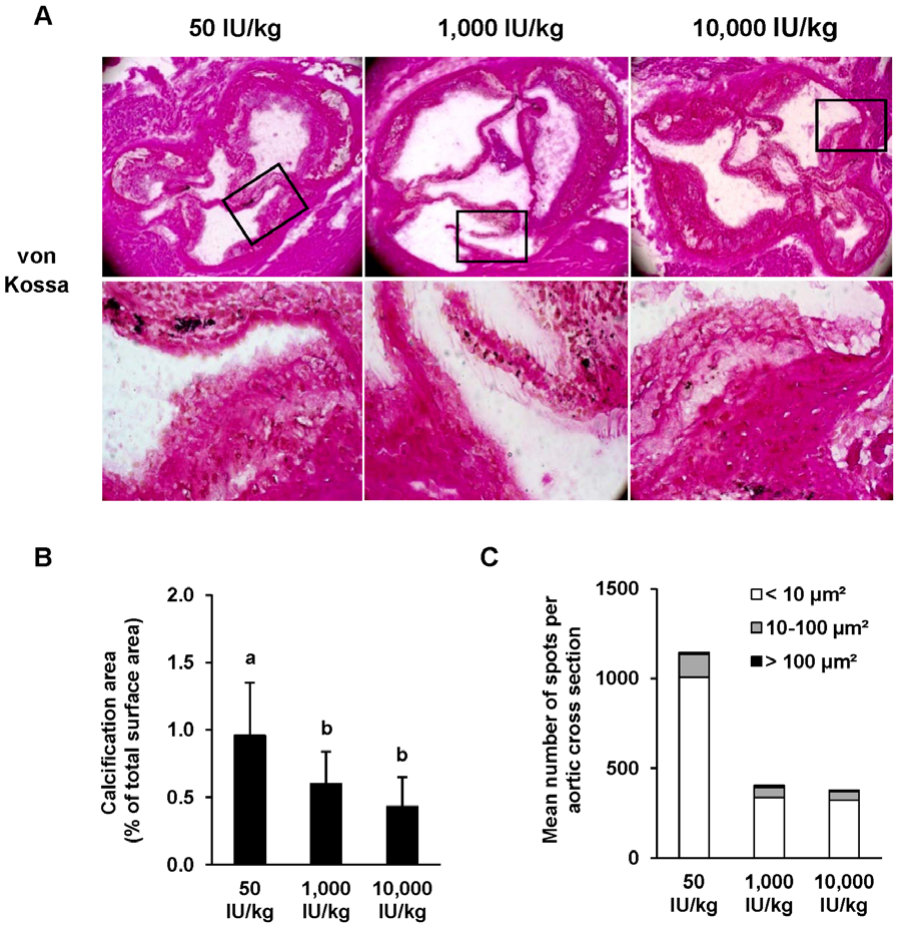
Low vitamin D diet stimulates the development of aortic calcification in LDLR^−/−^ mice. Panel A shows representative von Kossa stained aortic sections from LDLR^−/−^ mice kept for 16 weeks on a *Western* diet with either low (50 IU/kg), recommended (1,000 IU/kg) or high (10,000 IU/kg) vitamin D_3_ levels (×10). The bottom panel shows a magnification of the inserts in the top panel. Panel B shows the relative calcification area of the aortic sections and panel C the number of small-, medium- and large-sized calcified areas within an aortic cross-section. Data are means ± SD of 12 mice per group. ^a,b^ Bars with different superscript letters differ, *P*<0.015.

Data revealed that mice of all groups developed severe atherosclerosis. Thirty-five to 38% of the aortic root section areas comprised of plaque lesions (Table 3). The level of vitamin D in the diet had no influence on the atherosclerotic plaque sizes (Table 3). However, aortic sections showed more calcified lesions in mice fed the low vitamin D diet than in mice fed recommended or high amounts of vitamin D (+58%, +120%, respectively, Figure 4; *P*<0.001). Mice treated with the high vitamin D diet tended to have less aortic calcification areas (−28%) than mice fed recommended amounts (*P*<0.10). Figure 4C demonstrate that LDLR^−/−^ mice that were fed the low vitamin D diet had a markedly higher number of calcified spots within the aortic cross section than LDLR−/− fed diets with recommended and high amounts of vitamin D. The increased number of calcified spots was mainly due to a higher number of small (<10 µm^2^) and medium (10–100 µm^2^) spots but not due to a higher number of large (>100 µm^2^) spots. Mean plaque levels of collagen, lipids, macrophages and smooth muscle cells were not different between the three groups of LDLR^−/−^ mice (Table 3).

**Table 3:** Aortic plaque area size and plaque composition of aortic roots in LDLR^−/−^ mice fed low, recommended or high amounts of vitamin D_3_ with their diets.

	Dietary vitamin D_3_ concentrations		
	50 IU/kg	1,000 IU/kg	10,000 IU/kg
Plaque area (% of aortic cross section)	34.8±7.5	36.1±8.5	37.6±6.0
Plaque collagen (%)	59.9±5.0	57.8±12.9	55.8±6.4
Plaque lipids (%)	11.3±2.3	11.4±1.8	11.5±2.4
Plaque macrophages (%)	33.1±8.7	31.4±4.0	28.8±5.4
Plaque smooth muscle cells (%)	7.95±2.53	9.80±2.99	7.91±3.46

Values are means ± SD (n = 12 per group).

#### Impact of vitamin D deficiency on osteoblast differentiation factors in aortic roots of the LDLR−/− mice

**Figure 5:**
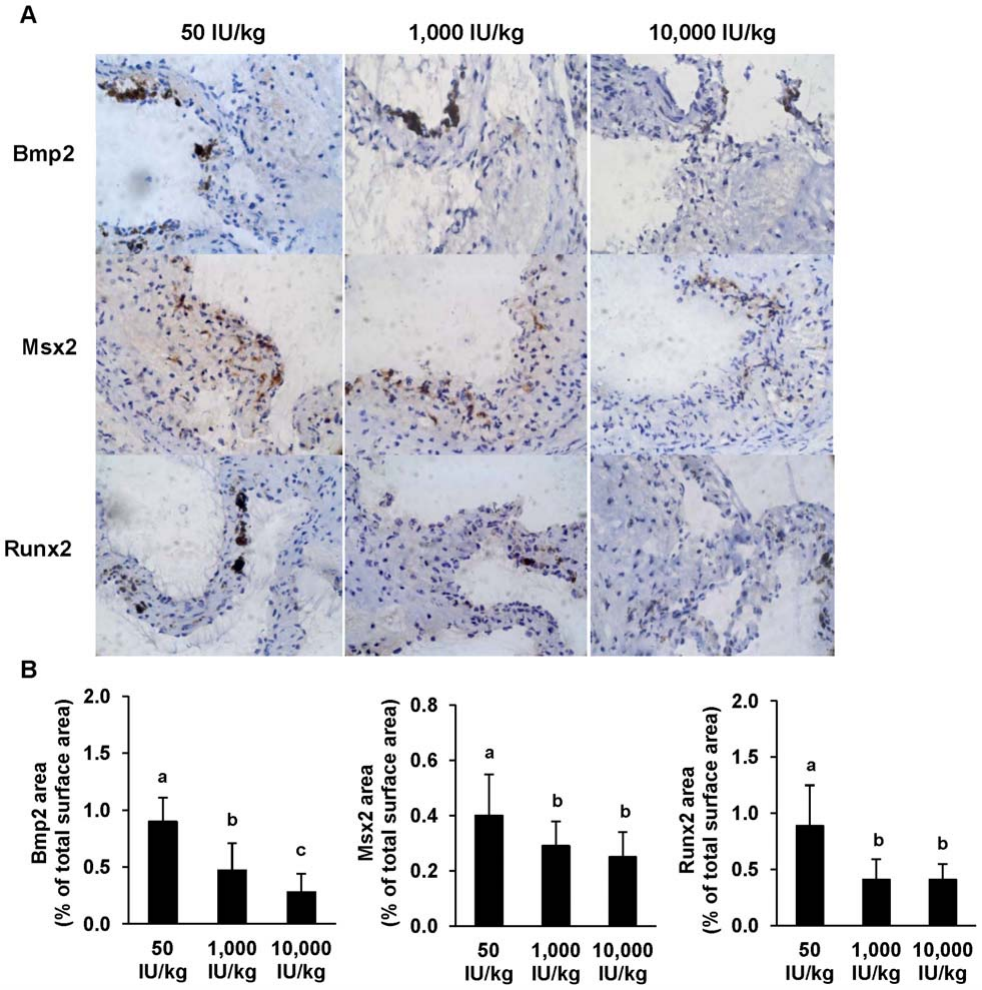
Low vitamin D diet increases the expression of vascular osteoblast differentiation factors in LDLR^−/−^ mice. Panel A shows representative aortic cross sections immunohistochemically stained for Bmp2, Msx2 and Runx2 from LDLR^−/−^ mice kept for 16 weeks on a *Western* diet with either low (50 IU/kg), recommended (1,000 IU/kg) or high (10,000 IU/kg) vitamin D_3_ levels (×10). Panel B shows the percentage of stained areas relative to the total cross-section. Data are means ± SD of 12 mice per group. ^a,b,c^ Bars with different superscript letters differ, *P*<0.015.

Since aortic calcification in the VDR^−/−^ mice was associated with the up-regulation of osteogenic key factors, we analysed these factors in the LDLR^−/−^ mouse model, too. Aortic Bmp2, Msx2 and Runx2 were higher in the low vitamin D group than in the groups fed recommended or high levels of vitamin D (*P*<0.01; Figure 5). Figure 5 shows further no differences of Msx2 and Runx2 between mice fed recommended and high levels of vitamin D, but mice fed the high vitamin D diet had lower Bmp2 levels than mice fed the recommended amounts (*P*<0.001).

## Discussion

Several studies found strong associations between hypovitaminosis D and cardiovascular diseases [4]–[8]. In the mouse experiments reported here, VDR knockout and diet-induced reduction of vitamin D status resulted in enhanced aortic valve and vascular calcification. Thus, the observed association of low serum 25(OH)D and high vascular calcifications in humans [15] appears to be causally linked. The results obtained from the VDR^−/−^ mouse study further revealed that calcification seems to be independent from other vascular processes because aortic valves of VDR^−/−^ mice became significantly calcified despite any identifiable atherosclerotic plaques. Vascular calcification is being associated with reduced arterial elastance, and compromised structural integrity [16], [17]. We assume that the observed vascular instability during preparation and dissection of aortas from VDR^−/−^ mice was, at least in part, caused by the presence of more calcified deposits at the level of aortic root compared to the WT mice. Not all vascular calcification occurs in the presence of atherosclerosis. Data from our VDR^−/−^ mouse study show that vascular mineralization was not simply the consequence of accelerated atherosclerosis because calcification occurred in vessel walls that were free of any atherosclerotic plaques. However, when considering the number of calcified spots of defined sizes within the cross sections, data clearly show that VDR^−/−^ had not only a higher number of small-sized spots, but also a higher number of medium- and large-sized spots compared to WT mice. Similar findings were observed in the LDLR^−/−^ mouse study, in which mice fed the low vitamin D diet had a higher number of small- and medium-sized calcified spots than mice fed recommended and high amounts of vitamin D. However, the number of large-sized spots was very low in all groups of these mice.

Analyses of human populations at high and moderate risk for coronary heart disease showed that circulating plasma concentrations of calcitriol were inversely correlated with the extent of vascular calcification in both groups [18]. Results from the LDLR^−/−^ mouse study revealed that mice fed the high vitamin D diet had less vascular calcification than mice fed the low vitamin D diet although plasma levels of 1,25(OH)_2_D_3_ were not different between these two groups. It is well-known that a high intake of vitamin D might lead to a reduction of plasma calcitriol levels via down-regulation of renal CYP27B1, an enzyme that converts 25(OH)D to 1,25(OH)_2_D [19]. We therefore assume that the described negative correlation between vascular calcification and plasma calcitriol only applies in cases of low plasma levels of calcitriol caused by chronic kidney diseases, low cutaneous vitamin D synthesis or low intake of dietary vitamin D but not in cases of reduced calcitriol levels induced by high vitamin D intake. Thus, intake of high doses of native vitamin D may differ in its effects on cardiovascular calcification when compared with high-doses of active vitamin D, i.e. calcitriol, since the levels of 1,25(OH)_2_D are not the same in these two states. Although the circulating concentrations of 25(OH)D, and 24,25(OH)_2_D and the vascular mRNA concentration of calbindin D_9 k_ reflect quite well the vitamin D status, levels beyond those obtained with recommended amount of dietary vitamin D were not associated with a further reduction of vascular calcification. Thus, the biphasic dose-response curve of vitamin D on vascular calcification may not necessarily be applicable to native dietary vitamin D.

Arterial calcification is a common feature of atherosclerosis [20], [21] and is supposed to contribute to an increased risk of vascular dissection during angioplasty [22], myocardial infarction [23] and mortality in patients with advanced atherosclerosis [24]. Vascular calcification is a complex process that involves numerous regulating factors and seems to share many similarities to skeletal mineralization and osteogenesis [25] because vascular smooth muscle cells and osteoblasts derive from the same mesenchymal precursor cell. Morphogenetic proteins Runx2, Msx2 and Bmp2 are involved in the transformation of vascular smooth muscle cells into osteoblast-like cells [26]–[28]. Results from our experiments indicate that the increased calcification of VDR^−/−^ mice and LDLR^−/−^ mice that received the low vitamin D diet was driven by these morphogenic proteins, because aortic Runx2, Msx2, and Bmp2 were up-regulated in these animals. Bmp2 has been identified as a crucial mediator of calcification because it up-regulates Msx2 and Runx2 which are key regulators of osteoblastic differentiation [29]. Runx2 expression in vascular smooth muscle cells has been considered identified as an early marker of osteoblastic differentiation, the initial step in vascular calcification [29]. Several factors that contribute to vascular calcification induce Runx2 expression, including increased inorganic phosphate concentration [30], [31]. The classical function of active vitamin D is to maintain the homeostasis of calcium and phosphorus and to keep their concentrations in the plasma constant. The findings that the circulating plasma concentration of inorganic phosphate did not differ between the VDR^−/−^ and WT mice, as well as among the LDLR−/− mice reveal that calcium and inorganic phosphate are not the crucial factors that determine vascular calcification in the vitamin D deficiency state.

In conclusion, our findings show that VDR deficiency and diets low in vitamin D promote aortic valve and aortic vessel calcification in the VDR^−/−^ and LDLR^−/−^ mouse models. The vascular calcification was associated with an up-regulation of osteoblast transcription factors which could have triggered the differentiation of vascular cells into osteoblast-like cells. We assume that an optimization of circulating 25(OH)D levels therefore could contribute to prevent vascular and aortic valve calcification and in consequence cardiovascular outcomes associated with those calcification processes.

## Materials and Methods

### Mice and Diets

All animal experiments described were carried out in accordance with established guidelines for the care and handling of laboratory animals and were approved by the council of Saxony-Anhalt (Permit number: 42502-5-34 MLU). Two studies were conducted. Animals were housed in cages on a 12-h-light/-dark cycle and consumed food and water *ad libitum*. Body weights and food intake were recorded weekly.

In the first experiment, male 5-weeks-old VDR^−/−^ mice (B6.129S4-Vdr^tmlMbd^/J; Jackson Laboratory, Bar Harbor, Maine, USA) and corresponding WT mice (C57BL/6J; Charles River Laboratories, Sulzfeld, Germany) were fed a semi-synthetic *Western* diet (20% sucrose, 20% coconut oil, 0.15% cholesterol) for 8 weeks. The VDR^−/−^ group (n = 6) was maintained on a high-calcium (2%), high-phosphorus (1.25%) rescue diet to prevent hypocalcaemia secondary to VDR deficiency. One group of WT mice (WT_RD_, n = 8) received the same rescue diet as the VDR^−/−^ group. The other group of WT mice (WT_ND_, n = 8) received a normal calcium/normal phosphorus diet (0.3% calcium, 0.156% phosphorus) according to the recommendations of the National Research Council (NRC) [32] Each group received 20% lactose with their diet to improve intestinal calcium uptake.

In the second experiment, a total of thirty-six 4-weeks-old male LDLR^−/−^ mice (B6.129S7-Ldlr^tm1Her^/J; Jackson Laboratory) were assigned to three groups of 12 mice each and were fed a semi-synthetic *Western* diet (20% sucrose, 21% lard, 0.15% cholesterol) containing either 50 (low), 1,000 (recommended [32]), or 10,000 (high) IU vitamin D_3_/kg diet for 16 weeks. The other vitamins and minerals were supplemented according to recommendations of the NRC [32].

### Sample Collection

Mice were deprived of food overnight prior to killing under light anesthesia with diethyl ether. Blood was collected into EDTA tubes. Plasma was separated by centrifugation at 1500×g for 20 min and stored at −20°C until further analysis. Aortic roots were harvested of all mice for histochemical and immunohistochemical analysis. From LDLR^−/−^ mice, femur was excised for calcium and phosphorus analysis. All tissue samples were immediately snap-frozen in liquid N_2_ and stored at −80°C.

### Analysis of Plasma 25(OH)D_3_, 24,25(OH)_2_D_3_ and 1,25(OH)_2_D_3_

Analysis of 25(OH)D_3_ and 24,25(OH)_2_D_3_ was done by a modified HPLC-MS/MS method [33] using an API 2000 (ABSCIEX, Darmstadt, Germany) in ESI mode coupled to an 1100 HPLC and Hypersil ODS column (100×2.1 mm^2^, 5 µm, Agilent Technologies, Waldbronn, Germany). Sample preparation was modified by mixing 50 µL of plasma and 50 µL of internal standard (25 µg/L 25(OH)D_3_-d_6_, Chemaphor Inc., Ottawa, Canada) in acetonitrile, followed by extraction with 400 µL n-hexane. After evaporation, 50 µL of 4-phenyl-1,2,4-triazoline-3,5-dione (0.15 g/L) in acetonitrile were added and incubated overnight. The samples were then mixed with 20 µL of ethanol, incubated for 30 min and dried under vacuum afterwards. The residues were dissolved in 40 µL of the mobile phase. Multilevel Serum Calibrator Set (Chromsystems, Munich, Germany) and horse serum spiked with 24,25(OH)_2_D_3_ (Enzo Life Sciences, Lörrach, Germany) were used for calibration. Plasma concentration of 1,25(OH)_2_D_3_ was determined by use of a commercial enzyme immunoassay (IDS, Boldon, UK).

### Analysis of Calcium and Phosphorus in Plasma and Femur

The concentration of calcium and total phosphorus (organic and inorganic) in plasma and femur was measured by inductively coupled plasma optical emission spectrometry (ICP-OES, Varian 715-ES ICP Optical Emission Spectrometer, Agilent Technologies, Santa Clara, California, USA; ICP Expert II Varian 715-ES Instrument Software, Version 1.1.3) using argon as carrier gas. Prior to analysis of calcium and phosphorus at selected wavelengths, femurs were ashed in a muffle furnace at 550°C and dissolved in HCl and HNO_3_ according to the official VDLUFA method [34]. After evaporation, the minerals were resolved in distilled water, filtered and injected in the ICP-OES system. The plasma concentration of inorganic phosphate was measured spectrophotometrically according to the manufacturers' protocol (Fluitest PHOS; Biocon Diagnostik, Vöhl/Marienhagen, Germany).

### Analysis of Triglycerides and Cholesterol in Plasma

Concentrations of triglycerides and cholesterol in plasma were examined using enzymatic reagent kits (DiaSys Diagnostic Systems, Holzheim, Germany, no. 1.1300 99 90 314 and 1.5760 99 90 314).

### Preparation and Morphometric Analysis of Aortic Roots

For preparation of the aortic root sections, the vasculature was perfused with 0.9% NaCl, and the ventricular edge and approximately 1 mm of the aortic root were immediately dissected under a stereomicroscope and cryo-mounted in mounting medium (tissue freezing medium®, Jung; Leica Instruments, Nussloch, Germany). To quantify atherosclerosis at the aortic root, serial 7 µm thick slices (CM 1850 UV microtome, Jung; Leica) were collected, beginning at the aortic valve area. Four sections each were stained with haematoxylin-eosin (sections from 0 to 28 µm), Mallory-Cason's trichrome for collagen structures (sections from 112 to 140 µm), von Kossa for vascular calcification (sections from 154 to 182 µm) and Oil red O for vascular lipids (sections from 196 to 224 µm). Histomorphological characterization and computerized morphometric quantification (Axiovert 200 microscope; AxioCamMRc; Axiovision Rel. 4.8.2 software (all Carl Zeiss, Jena, Germany)) of the atherosclerotic lesions were performed. The cross-sectional surface area of the total vessel, the atherosclerotic lesion area as well as the calcification area, the collagen area and the lipid area were assessed to characterize atherosclerosis development in the aortic root.

### Immunohistochemical Analysis of Aortic Roots

For immunohistochemistry aortic root sections were immediately fixed in acetone at −20°C for 10 min, and endogenous peroxidase was blocked with 0.3% H_2_O_2_ in methanol. Following a washing step, the sections were incubated each with 5% blocking serum in PBS at room temperature for 20 min. Sections were incubated with primary antibodies against CD68 (AbD Serotec, Oxford, UK; sections from 28 to 56 µm), SMC α-actin (Sigma-Aldrich, Taufkirchen, Germany; sections from 56 to 84 µm), Bmp2 (abcam, Cambridge, UK; sections from 84 to 112 µm), Runx2 (antibodies-online.de, Atlanta, USA; sections from 140 to 154 µm ) and Msx2 (Sigma-Aldrich; sections from 182 to 196 µm) over night at 4°C, followed by incubation with horseradish peroxidase-labeled secondary antibodies (goat anti-rat IgG (AbDSerotec, Oxford, UK) and rabbit anti-mouse IgG (Dako, Hamburg, Germany) at room temperature for 30 min. The immunocomplex was visualized using diaminobenzidine chromogen (Dako). Sections were counterstained with Harris haematoxylin solution. Stained slices were evaluated for the presence and intensity of positive reaction using Axiovision Rel. 4.8.2 software.

### RNA Isolation and real time RT-PCR

For the determination of aortic calbindin D_9 k_ mRNA expression level, total RNA was isolated from the aortic arch using TrizolTM reagent (Invitrogen, Karlsruhe, Germany) according to the manufacturer's protocol. RNA concentration and purity were estimated from the optical density at 260 and 280 nm, respectively. Total RNA (1.2 µg) was subjected to first-strand cDNA synthesis at 42°C for 60 min using M-MuLV RT (MBI Fermentas, St. Leon-Rot, Germany) and oligo dT18-primer (Operon Biotechnologies, Cologne, Germany). PCR was performed with the Rotor Gene 2000 system (Corbett Research, Mortlake, Australien). Real-time detection was carried out by using SYBR® Green I (Sigma), 1.25 U Taq DNA polymerase (Promega, Mannheim, Germany), 500 µM dNTP and 26.7 pmol of specific primers for calbindin D_9 k_ (NM_009789.2; forward 5′TCA CCT GCT GTT CCT GTC TG 3′; reverse 5′ TCG CCA TTC TTA TCC AGC TC 3′, Operon Biotechnologies). Each PCR cycle compromised denaturation for 20 s at 95°C, annealing for 30 s at 60°C and elongation at 72°C for 55 s. At the end of the elongation step, the intensity of fluorescence in all probes was measured. The threshold cycle (C_t_) and the amplification efficiency were obtained from each amplification curve using the software RotorGene 4.6 (Corbett Research). For identification of the PCR products, melting-curve analysis and agarose (1.5%) gel electrophoresis were performed. The housekeeping gene β-actin (NM_007393.2; forward 5′ACG GCC AGG TCA TCA CTA TTG 3′; reverse 5′ CAC AGG ATT CCA TAC CCA AGA AG 3′, Operon Biotechnologies) was used for normalization.

### Statistical Analysis

Values presented in the text are means and standard deviations. Treatment effects were analyzed by one-way ANOVA using MINITAB software (Minitab, State College, PA, USA). For statistically significant F values, means were compared by Fisher's multiple-comparison test. Means were considered to be significantly different at *P*<0.05.
